# Persistent blood DNA methylation changes one year after SARS-CoV-2 infection

**DOI:** 10.1186/s13148-022-01313-8

**Published:** 2022-07-23

**Authors:** Joseph Balnis, Andy Madrid, Kirk J. Hogan, Lisa A. Drake, Anish Adhikari, Rachel Vancavage, Harold A. Singer, Reid S. Alisch, Ariel Jaitovich

**Affiliations:** 1 Division of Pulmonary and Critical Care Medicine, Albany Medical College, Albany, USA; 2 Department of Molecular and Cellular Physiology, Albany Medical College, 47 New Scotland Avenue, MC91, Albany, New York 12208 USA; 3 Department of Neurological Surgery, Albany Medical College, Madison, Wisconsin, USA; 4 Department of Anesthesiology, University of Wisconsin School of Medicine and Public Health, Madison, WI USA

**Keywords:** COVID-19, DNA methylation, SARS, PASC

## Abstract

We recently reported the COVID-19-induced circulating leukocytes DNA methylation profile. Here, we hypothesized that some of these genes would persist differentially methylated after disease resolution. Fifteen participants previously hospitalized for SARS-CoV-2 infection were epityped one year after discharge. Of the 1505 acute illness-induced differentially methylated regions (DMRs) previously identified, we found 71 regions with persisted differentially methylated, with an average of 7 serial CpG positions per DMR. Sixty-four DMRs persisted hypermethylated, and 7 DMR persisted hypomethylated. These data are the first reported evidence that DNA methylation changes in circulating leukocytes endure long after recovery from acute illness.

**Supplementary Information:**

The online version contains supplementary material available at 10.1186/s13148-022-01313-8.

## Introduction

The COVID-19 pandemic has caused 6 million deaths worldwide. Many COVID-19 survivors fail to recover their pre-infection status, with lasting physical impairments and increased risk of cardiovascular events [1]. The pathophysiology of Post-Acute Sequelae of SARS-CoV-2 Infection (PASC) is poorly understood, and instruments commonly used in clinical practice to assess organ function fail to correlate with patient-reported symptoms. Identification of biological mechanisms underpinning persistent deficits will accelerate research to better understand, predict, and manage PASC. Because an organism’s cells share identical genetic information, different phenotypes are established and maintained by epigenetic mechanisms [2]. DNA methylation is a covalent yet dynamic epigenetic modification that influences gene expression profiles, especially when present in gene promoter regions [2]. Differentially methylated regions (DMRs) comprise serial cytosine–guanine dinucleotide (CpG) positions that are consecutively hyper- or hypo-methylated and can persist over long periods of time [3]. Accordingly, DNA methylation is a plausible mechanism to maintain an abnormal cellular phenotype after resolution of acute disease. Because PASC is caused by prior SARS-CoV-2 infection and host inflammatory responses, circulating leukocytes are attractive targets to investigate differential DNA methylation induced by acute infection. We have shown that SARS-CoV-2 infection disrupts the circulating leukocyte DNA methylome [4] and transcriptome [5] in correlation with disease severity spanning full recovery to death. We found that SARS-CoV-2 infection is characterized by 1505 DMRs compared to healthy control individuals, and gene ontological analysis indicates that these genes participate in immune responses, leukocyte activation, viral responses, and related processes. Thus, we reasoned that a subset of these SARS-CoV-2 DMRs could endure long after recovery from COVID-19.

## Methods

**Fig. 1:**
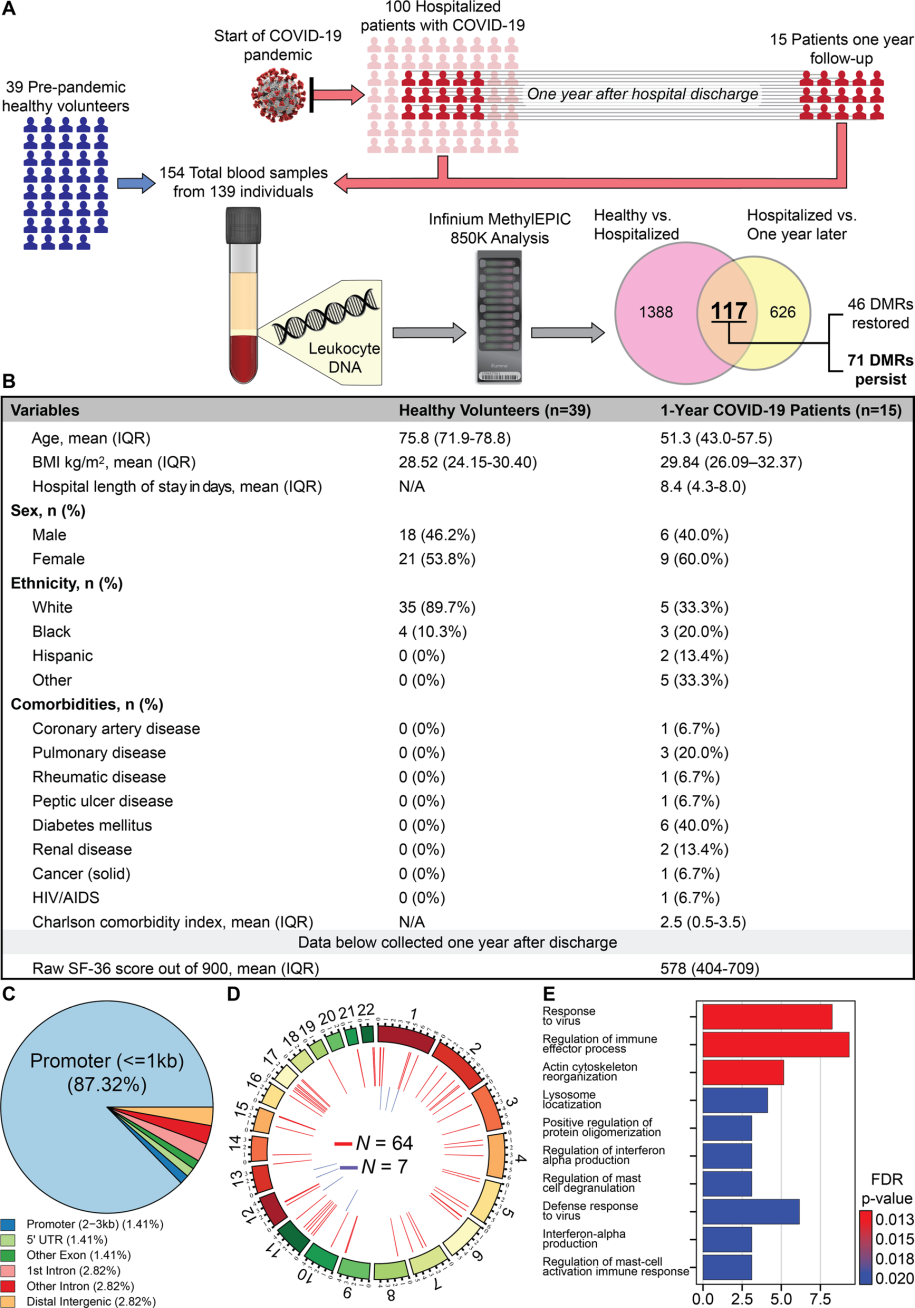
**A** Diagram of data generation and analysis pipeline. See text for details. **B** Clinical characteristics of participants. To prevent DNA methylation changes caused by asymptomatic SARS-CoV-2 infection, samples were taken from healthy volunteers enrolled in 2017, who were not recalled. IQR is interquartile range. Raw SF-36: Short Form Health Survey involves 36 questions that are divided in 9 domains. Each domain has a maximal score of 100% based on the participants answers, and thus, the optimal score is 900. **C** Pie chart showing the distribution of DMRs to standard genomic features in percent. 5′UTR=5′ untranslated region. In keeping with the known role of DNA methylation in regulation of gene expression, a preponderance of DMRs is in gene promoter regions. **D** Circos plot shows the genomic distribution of differentially methylated regions (DMRs) across the human genome (outer ring). Each chromosome is shown as a different color. Relative chromosome size is denoted by the arc bar length (inner rings). Hyper-methylated DMRs are shown in red, and hypo-methylated regions are shown in blue. Sex chromosomes were omitted from the analysis. These results indicate that 71 DNA regions persist differentially methylated one year after hospital discharge in reference to a pre-pandemic healthy control cohort. **E** Bar graph of the top 10 gene ontological (GO) processes related to the SARS-CoV-2-associated differentially methylated genes that persist abnormal one year after hospital discharge ordered by statistical significance. The X-axis provides the number of SARS-CoV-2 DMR-associated genes that contribute to each GO term. Bar color indicates the FDR P-value by using a Fischer test. These results indicate that the observed DMRs occur in genes that participate in process such as response to virus, regulation of immune processes and others.

To investigate this hypothesis, all the participants from our original cohort who survived COVID-19 hospitalization between March and April 2020 were recontacted 1 year after discharge (Fig. 1A, B). Specific description of the cohort can be found in previous publications [4–6]. Fifteen patients out of the original 102 participants and corresponding to 30% of surviving individuals consented to a second office visit for clinical evaluation and a new blood sample for further analysis. Upon evaluation, these patients expressed multiple PASC symptoms including fatigue, sleep disturbances, and reduced general heath scores. However, they denied dyspnea and showed normal hemoglobin oxygen saturation while breathing ambient air. Leukocyte DNA was purified and bisulfite-converted for DNA methylation analysis using the Infinium Human MethylationEPIC 850K BeadChip on an Illumina^®^ platform. Genome-wide leukocyte DNA methylation status was compared to samples from 39 healthy volunteers which were analyzed with the same platform as previously reported [4]. These healthy volunteers were enrolled before the current pandemic, ruling out possible differential DNA methylation caused by asymptomatic COVID-19 infection, and were older than the SARS-CoV-2 patients (78 vs 51 years old, respectively); other characteristics are presented in Fig. 1B. The specific comparisons made to identify the persistent DMRs are shown by the Venn diagram in panel A of the figure. To adjust for batch effects, and given that these were patient-matched specimens, the model used for differential methylation was adjusted for patient ID. Following model selection, R packages ComBat and SVA were employed to adjust for known batch effects and latent confounding variables, respectively, and were adjusted for in the model. Differential methylation analysis was then performed as recently reported [4].

## Results

Of the 1505 acute illness induced DMRs we previously identified [4], 71 DMRs persisted significantly differentially methylated 1 year thereafter, with an average of 7 serial CpG positions per DMR. Sixty-four DMRs persisted hypermethylated, and 7 DMR persisted hypomethylated (*p *< 0.0001). Over 90 % of the lasting DMRs were located near or within gene promoter regions (Fig. 1C), suggesting an effect on gene expression regulation [2]. DMRs were uniformly distributed along the entire genome (Fig. 1D). Gene ontological (GO) enrichment analysis of the genes harboring the lasting DMRs included pathways related to viral responses and inflammation (Fig. 1E), see also accession numbers GSE174818 and GSE197152. For details regarding the specific genes that persist dysregulated one year after hospital discharge and their corresponding chromosomal location, see Additional file 1: Table S1.

## Discussion

More than 6 million deaths have been attributed to COVID-19, primarily arising from acute respiratory failure [7]. Recent data indicate that disease severity predominantly depends on host factors [8, 9], supporting the need to better differentiate individual responses at the molecular level. We and others have described outcome-specific multi-omic profiles of COVID-19 patients [4, 5, 10]. However, specific host mechanisms that coordinate expression of these profiles are unresolved. While an individual’s nucleated cells share identical genomic sequences, distinct cellular phenotypes are established and maintained by epigenetic mechanisms [11, 12], including DNA methylation, histone and chromatin modifications, and non-coding RNA transcription [2]. DNA methylation regulates gene expression and is sensitive to environmental factors [2, 13–17]. Methylation of CpGs located in promoter regions is canonically associated with transcriptional repression [2]. Mechanistically, methylated CpGs recruit complexes containing methyl-CpG binding domain proteins and other factors that aggregate into multiprotein repressive complexes to silence transcription [18, 19]. Critically ill patients have altered circulating blood DNA methylation profiles [20, 21], consistent with epigenetic regulation of gene expression. We have recently reported a genome-wide DNA methylation analysis of patients with COVID-19 in correlation with clinical outcomes spanning full recovery to death, and multiple sources have reported that DNA methylation is relevant in the pathophysiology of acute COVID-19 infection [22–24]. These findings introduce evidence of acute epigenetic regulation of genes associated with COVID-19 severity [4]. Although many patients who survive COVID-19 develop long-term cognitive and somatic dysfunctions [25], no pathobiological processes that account for these lingering deficits have been identified. We present here evidence that epigenetic marks can persist beyond clinical resolution of acute illness. These data are the first reported evidence that DNA methylation changes in circulating leukocytes endure at least 1 year after recovery from acute COVID-19 illness, leaving durable marks in the methylome that may condition patterns of gene expression that drive PASC pathophysiology. Accordingly, DNA methylation may be a mechanism regulating leukocyte adhesion and vascular injury and contribute to the recently described higher risk of cardiovascular events after COVID-19 [1]. A limitation of our study is that the age difference between the healthy, pre-pandemic, and the SARS-CoV-2 cohorts. Interestingly, recent evidence indicates that epigenetic clocks are not accelerated by acute COVID-19 infection [26], and the comparison between epigenetic and chronological ages in our cohort has been found not significant [4]. Other limitations of this study include the use of a DNA methylation detection platform that targets a limited number of CpGs (~4% of CpGs in the entire methylome) and the relatively small cohort size. Future studies comprising larger cohorts and whole-genome methylation and RNA sequencing may serve to further identify regions and transcripts that associate with, and predict, PASC phenotypes and that contribute to disabling COVID-19 sequelae.

## Supplementary Information

**Additional file 1.** List of genes persisting dysregulated one year after hospital discharge.

## Data Availability

The datasets generated during and/or analyzed during the current study are publicly available at GSE174818 and GSE197152.
